# Geochemical evidence for the internal migration of gas condensate in a major unconventional tight petroleum system

**DOI:** 10.1038/s41598-022-11963-6

**Published:** 2022-05-13

**Authors:** James M. Wood, Jaime Cesar, Omid H. Ardakani, Arka Rudra, Hamed Sanei

**Affiliations:** 1 Calaber1 Resources, 965 Sierra Morena Court SW, Calgary, AB T3H 3R3 Canada; 2 Natural Resources Canada, Geological Survey of Canada, 3303 33rd Street NW, Calgary, AB T2L 2A7 Canada; 3 Department of Geoscience, University of Calgary, 2500 University Dr. NW, Calgary, AB T2N 1N4 Canada; 4 Lithospheric Organic Carbon (LOC) Group, Department of Geoscience, Aarhus University, Høegh-Guldbergs Gade 2, 8000C Aarhus, Denmark

**Keywords:** Climate sciences, Solid Earth sciences, Energy science and technology

## Abstract

Unconventional petroleum systems go through multiple episodes of internal hydrocarbon migration in response to evolving temperature and pressure conditions during burial and uplift. Migrated fluid signatures can be recognized using stable carbon isotope and PVT compositional data from produced samples representative of in-situ petroleum fluids. Such samples, however, are seldom collected due to operational complexity and high cost. Here, we use carbon isotope and PVT data from co-produced hydrocarbon gas and liquid to provide evidence for widespread migration of gas-condensate in the Montney unconventional petroleum system of western Canada. Extended C_1_–C_33_ isotopic profiles exhibit convex upward signatures with C_4_–C_5_ maxima at low molecular weight, and increasing or nearly uniform signatures at high molecular weight. Additionally, recombination PVT compositional data show C_6_–C_15_ condensate concentrations are higher than expected for unmodified oils. The combined convex upward and increasing or uniform isotopic signatures are interpreted as mixing profiles formed by the introduction of high-maturity gas-condensate (C_1_–C_15_) to shallower zones with in-situ hydrocarbon fluids of lower thermal maturity. The recognition of widespread gas-condensate migration adds to the complex history of internal hydrocarbon migration within the Montney tight-petroleum system including previously identified migration episodes of early oil and late-stage methane-rich gas.

## Introduction

**Figure 1:**
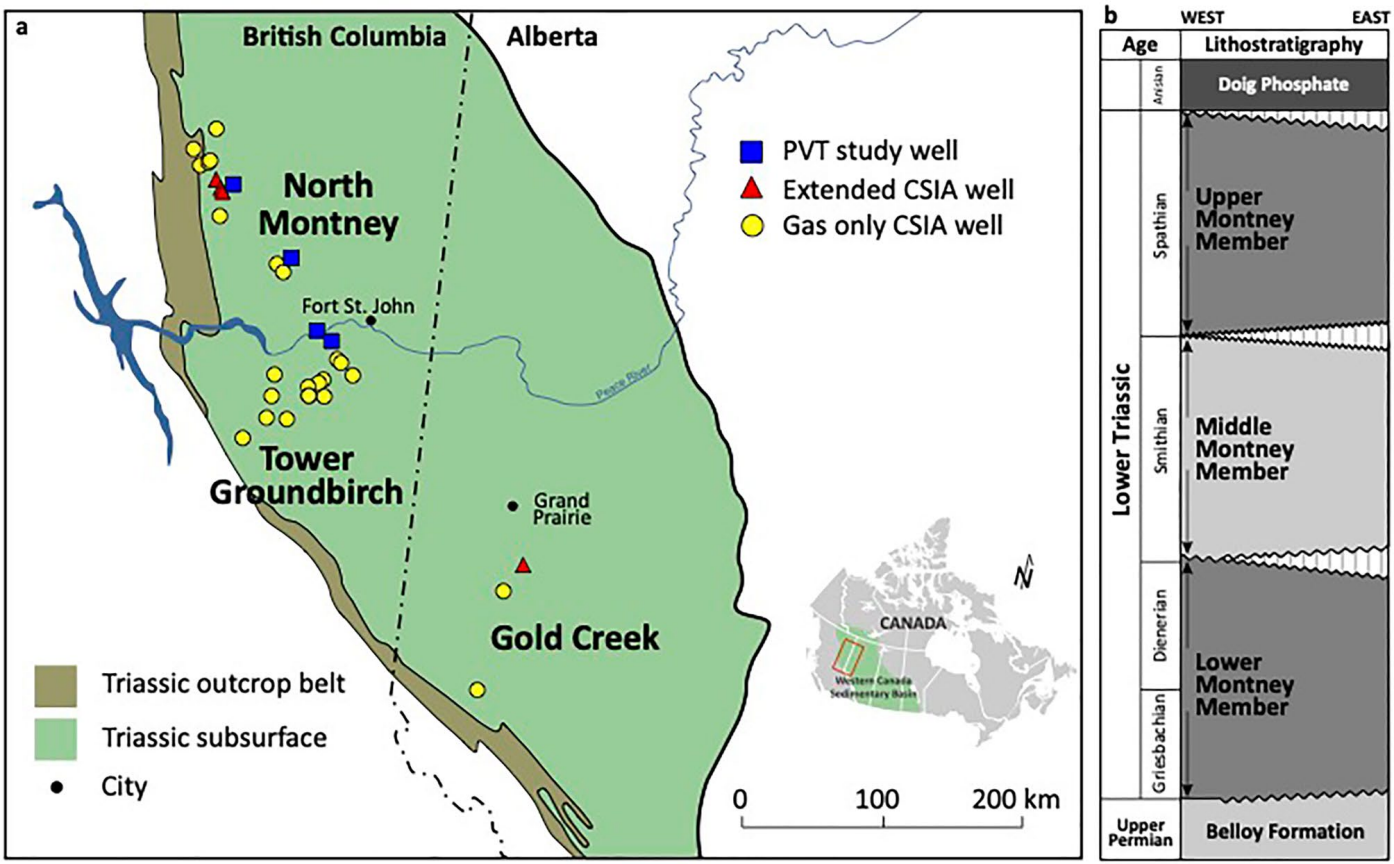
Geographic and stratigraphic setting of the Montney unconventional petroleum system. (**a**) Map showing the location of the Montney unconventional petroleum system and the three geographic study areas at North Montney, Tower-Groundbirch and Gold Creek. Symbols show locations of wells with data used in this study; *PVT* recombination PVT compositional data, *CSIA* compound-specific isotope analysis. Modified from Zelazny et al.^69^. Used with permission of CSPG. (**b**) Stratigraphic column showing relationships of the Montney Formation and its three main constituent members (Lower, Middle, Upper). Modified from Zonneveld and Moslow^28^. Used with permission of CSPG.

The distribution of hydrocarbon fluids in many low-permeability (“tight”) unconventional petroleum systems is significantly influenced by the internal (intraformational) migration of hydrocarbons. This in turn can lead to economic impacts on petroleum production such as higher gas-oil ratios and lower hydrocarbon liquid contents than expected from routine thermal maturity proxies^1–7^. Internal migration of hydrocarbons in the siltstone-dominated Montney tight-petroleum system of the Western Canadian Sedimentary Basin (WCSB) has been increasingly recognized in recent years, particularly the late-stage migration of methane-rich gas^8–14^ largely during basin uplift^15–17^. Additionally, there is evidence for an early episode of internal oil migration from distal marine source rocks to more proximal reservoir siltstones^18–20^. Here, we use compound-specific isotope analysis (CSIA) and recombination PVT studies of produced hydrocarbon gases and liquids from three geographic study areas (North Montney, Tower-Groundbirch and Gold Creek; Fig. 1a) to show that an additional widespread episode of condensate-range hydrocarbon migration occurred within the Montney tight-petroleum system. The recognition of internal migration of gas-condensate sheds new light on the present-day distribution of hydrocarbon fluids within the Montney Formation, and will assist future economic evaluation and exploitation of this world-class tight-petroleum resource. Multiple episodes of internal hydrocarbon migration are likely to have occurred in many other unconventional tight-petroleum systems globally, and the Montney serves as a valuable analogue. Migration and leakage of gas from unconventional tight-petroleum basins, such as the Montney, results in up-dip transmission of methane from deep accumulations to shallower zones, and ultimately the atmosphere with potential for significant natural greenhouse gas emissions^15^.

## Geologic setting

**Figure 2:**
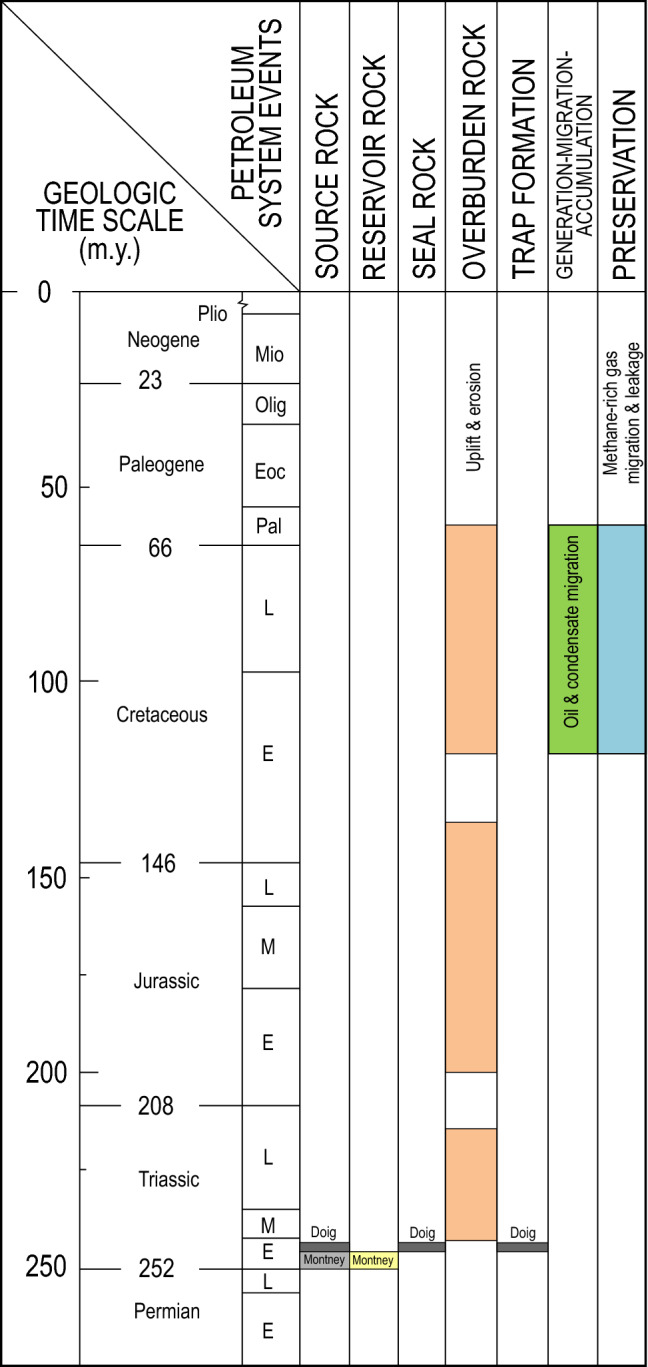
Petroleum system events chart^29^ for the Montney-Doig unconventional tight-petroleum system. Events are based mainly on information from Henderson et al.^27^, Ness^30^, and Ducros et al.^31^. Overburden rock, generation-migration-accumulation and preservation events are tied to timing of burial and uplift which varies significantly with geographic position in the Western Canadian Sedimentary Basin^30,31^. *m.y.* million years, *E* early, *M* middle, *L* late.

The Montney Formation hosts a world-class resource of unconventional tight gas and hydrocarbon liquid located directly northeast of the Cordilleran deformation belt in the WCSB of British Columbia and Alberta^8,14,21,22^. Marketable unconventional petroleum resources (i.e., petroleum recoverable under foreseeable economic and technological conditions) in the Montney have been estimated at 449 Tcf of natural gas, 14,521 million barrels of natural gas liquids and 1125 million barrels of oil^22^. The present-day extent of the Montney Formation is bounded by an erosional subcrop edge to the northeast and the Cordilleran deformation belt to the southwest (Fig. 1a). The formation unconformably overlies the Permian Belloy Formation, and is unconformably overlain by the Triassic Sunset Prairie Formation or Doig Phosphate Zone (Fig. 1b). It forms a mixed clastic-carbonate sedimentary wedge that thickens from less than 1 m close to the subcrop edge in the northeast in Alberta to over 400 m in the southwest in British Columbia^13,23,24^. Montney rocks in the unconventional tight-petroleum trend are composed mainly of medium- to coarse-grained, dolomitic to subarkosic siltstones that were deposited in lower shoreface to offshore environments along the western margin of the Pangea supercontinent during the Early Triassic (ca. 252–247 Ma, Fig. 2)^23–28^. The typical dolomitic-feldspathic composition and silt grade of Montney rocks suggest arid continental source areas with limited chemical weathering, and sediment delivery to marine environments by aeolian processes and ephemeral rivers^23,28^. The Montney Formation is divided into three main members (Lower, Middle and Upper) that each coincides with a third-order unconformity-bounded stratigraphic sequence (Fig. 1b). These three sequences are globally recognized and coincide with the Griesbachian-Dienerian, Smithian and Spathian substages of the Early Triassic^23,24,27,28^.

The unconventional Montney–Doig petroleum system had a complex geologic history which is simplified and summarized in the petroleum system events chart^29^ presented in Fig. 2. The Cordilleran Orogeny, which began in mid-Jurassic time, resulted in thrust sheet loading, and the establishment of a foreland basin in front of the rising orogen. The foreland basin underwent rapid subsidence, and the Montney and adjacent formations started to generate oil at approximately 90–135 Ma^30^ (Fig. 2). With further burial to maximum depths of 4–7 km at 60–80 Ma^30,31^, and the accompanying increase in temperature, oil fractions cracked forming solid bitumen, condensate and gas, and significant over-pressuring developed^32,33^. From maximum burial to the present-day, the Montney tight-petroleum system underwent cooling, cessation of hydrocarbon generation, depressurization, and stress reduction in response to basin uplift, relaxation of tectonic compression, and erosion of 1.4–3.0 km of overburden^30,31^ (Fig. 2). Natural leakage of hydrocarbon fluids during uplift (Fig. 2) contributed significantly to depressurization of the unconventional Montney petroleum system, and was dominated by natural gas, which was preferentially enriched in methane by various processes including phase separation and secondary migration^15,17^.

Unconventional reservoir rocks in the Montney Formation form part of the greater Triassic petroleum system of the WCSB^34^. The highest quality source rocks in the Triassic petroleum system are organic-rich phosphatic rocks in the basal portion of the Doig Formation (Fig. 2), which unconformably overlies the Montney Formation (Fig. 1b) across most of the unconventional tight-petroleum fairway^13,34–37^. Doig source rocks, with TOC content of 1.1–9.5 wt% and dominantly Type II kerogen, are thought to have generated significant volumes of oil, which migrated into adjacent Triassic formations including the Montney^35,37^ (Fig. 2). In up-dip, conventional portions of the Montney in Alberta, oil likely also migrated into the formation from unconformably overlying Jurassic source rocks^35,37^. A contribution of self-sourced oil from organic-rich mudstone facies within the Montney Formation was initially contemplated by Riediger^36^. Subsequent regional Montney organic petrography and pyrolysis work in Alberta^38^ documented multiple organic maceral types including both primary sedimentary organic matter (primarily alginite) and secondary organic matter (primarily solid bitumen). However, the TOC content of Alberta Montney rocks is low, typically ranging from 0.3 to 1.5 wt%^38^, and oil generated internally may have been insufficient to fully charge the Montney Formation. Montney rocks in northeast British Columbia typically have higher TOC content (0.3–4.0 wt%) than those in Alberta, and the organic matter is dominantly in the form of solid bitumen or pyrobitumen^32,39–43^. The solid bitumen represents a previous pore-filling oil phase, and has a negative influence on petrophysical parameters that control fluid flow and deliverability such as pore throat size and matrix permeability^41,44,45^. Self-sourced Montney oil may have undergone both local-scale migration and long-range lateral migration. Oil generated in thin mudstone intervals is interpreted to have migrated locally into adjacent Montney siltstone intervals^46^. Additionally, oil generated in distal marine organic-rich source rocks to the southwest is thought to have migrated laterally updip into shallower Montney zones^18–20^.

The Montney Formation contains three main present-day hydrodynamic systems that conform broadly with structural elevation: (1) a deep, over-pressured, gas-dominated unconventional system in the southwest, (2) an under-pressured, oil-dominated unconventional system at intermediate depth, and (3) a shallow, normally-pressured conventional system in the northeast with stratigraphically and structurally trapped oil and gas^14^. The over-pressured, gas-dominated and under-pressured oil-dominated systems together comprise an unconventional petroleum system (basin-centered system)^47^ characterized by regionally pervasive hydrocarbons, low permeability, absence of a down-dip water contact, and absence of conventional seals and traps. Present-day oil, condensate, wet gas and dry gas reservoir-fluid windows in the Montney tight-petroleum system are arranged generally in accord with increasing depth and thermal maturity to the southwest, and extend regionally for 10 s of kilometers in the dip direction and 100 s of kilometers along strike^8,12,13^. The composition and distribution of Montney hydrocarbon fluids determined by thermal maturity at maximum burial were significantly modified during basin uplift by the secondary migration of methane-rich gas (Fig. 2) along intricate, stratigraphically and structurally controlled fairways^9–13,15–17^.

## Results

### Carbon isotope ratios of produced gas

**Figure 3:**
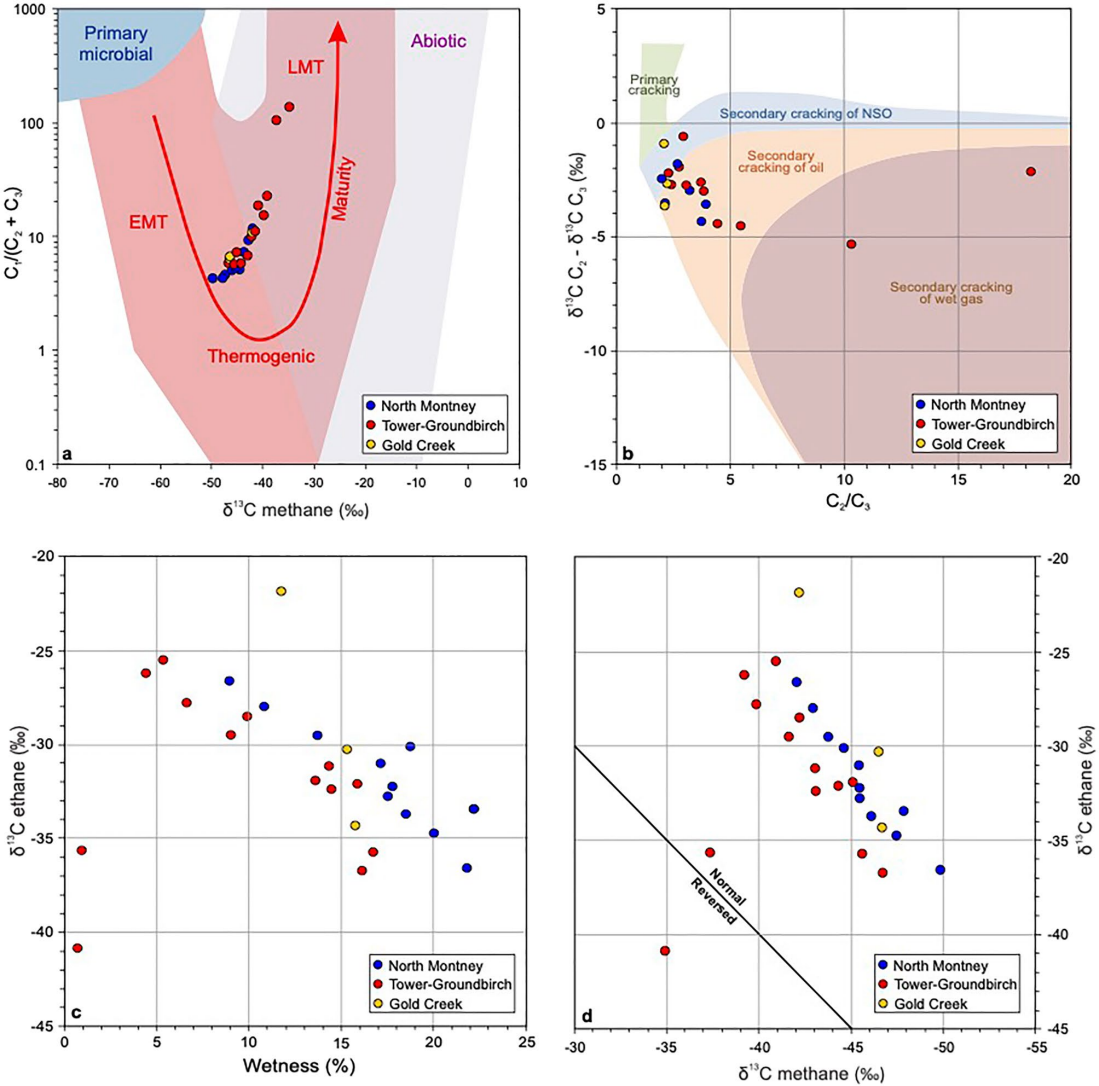
Compositional and isotopic plots for Montney produced gas samples. (**a**) δ^13^C methane vs. C_1_/(C_2_ + C_3_) Bernard plot^48^. Gas genetic fields and maturity trend (red arrow) from Milkov and Etiope^49^: *EMT* early mature thermogenic gas, *LMT* late mature thermogenic gas. (**b**) C_2_/C_3_ vs. δ^13^C ethane—δ^13^C propane Lorant plot^50^. NSO signifies petroleum fluid rich in polar nitrogen-, sulphur- and oxygen-containing organic compounds. (**c**) Gas wetness (Σ(C_2_–C_5_)/Σ(C_1_–C_5_)) vs. δ^13^C ethane cross-plot. (**d**) δ^13^C methane vs. δ^13^C ethane cross-plot. In panel (**a**), Montney produced gas samples from the three widely separated geographic areas (Fig. 1a) form a coherent maturity trend of oil-associated thermogenic gases. In panel (**b**), Montney produced gas samples lie in fields expected for secondary cracking of oil and wet gas. In panels (**c**,**d**), the majority of the samples form a normal maturity trend, whereas two samples from the Groundbirch area indicate an ethane isotopic rollover/reversal zone^51,52^.

Carbon isotope ratio and compositional data for 27 produced gas samples from Montney wells located in three separate geographic areas (Fig. 1a) are listed in Supplementary Tables S1 and S2. A Bernard plot^48^ of δ^13^C methane vs. C_1_/(C_2_ + C_3_) (Fig. 3a), constructed using these data, shows that most of the Montney produced gas samples are clustered along a linear maturity trend in the middle portion of the oil-associated thermogenic gas genetic field^49^. Two samples from the Groundbirch area (Fig. 1a) lie on the same trend but at higher maturity in the late mature thermogenic gas genetic field^49^. A Lorant plot^50^ of C_2_/C_3_ versus δ^13^C ethane–δ^13^C propane (Fig. 3b) shows the Montney produced gas samples lie in the fields expected for secondary cracking of oil and wet gas.

A gas wetness vs. δ^13^C ethane cross-plot (Fig. 3c) indicates that all except two gas samples lie on a normal thermal maturity trend with δ^13^C ethane increasing as gas wetness (Σ(C_2_–C_5_)/Σ(C_1_–C_5_)) decreases. The two gas samples with lowest wetness values (highest methane contents) are both from the deepest southwest portion of the Groundbirch area, and are consistent with rollover and reversal of δ^13^C ethane values^51^. A δ^13^C methane vs. δ^13^C ethane cross-plot (Fig. 3d) confirms these two gas samples are in the δ^13^C ethane rollover and reversal zone^51^. All the other gas samples show a positive correlation of δ^13^C methane and δ^13^C ethane values, consistent with a normal thermal maturity trend. In this context, a normal maturity trend has δ^13^C values with C_1_ < C_2_ < C_3_ whereas an isotope reversal has C_1_ > C_2_ > C_3_ (full reversal) or C_1_ > C_2_ < C_3_ (partial reversal)^52^. A normal maturity trend is also characterized by a decrease in gas wetness and an increase in δ^13^C values of C_1_–C_5_ components with increasing maturity^51,52^. An isotope rollover occurs at high maturity when the normal maturity trend ‘‘rolls over” with increasing gas wetness and the δ^13^C values of gas components (e.g., ethane or propane) decrease becoming isotopically lighter (Fig. 3c)^51,52^.

**Figure 4:**
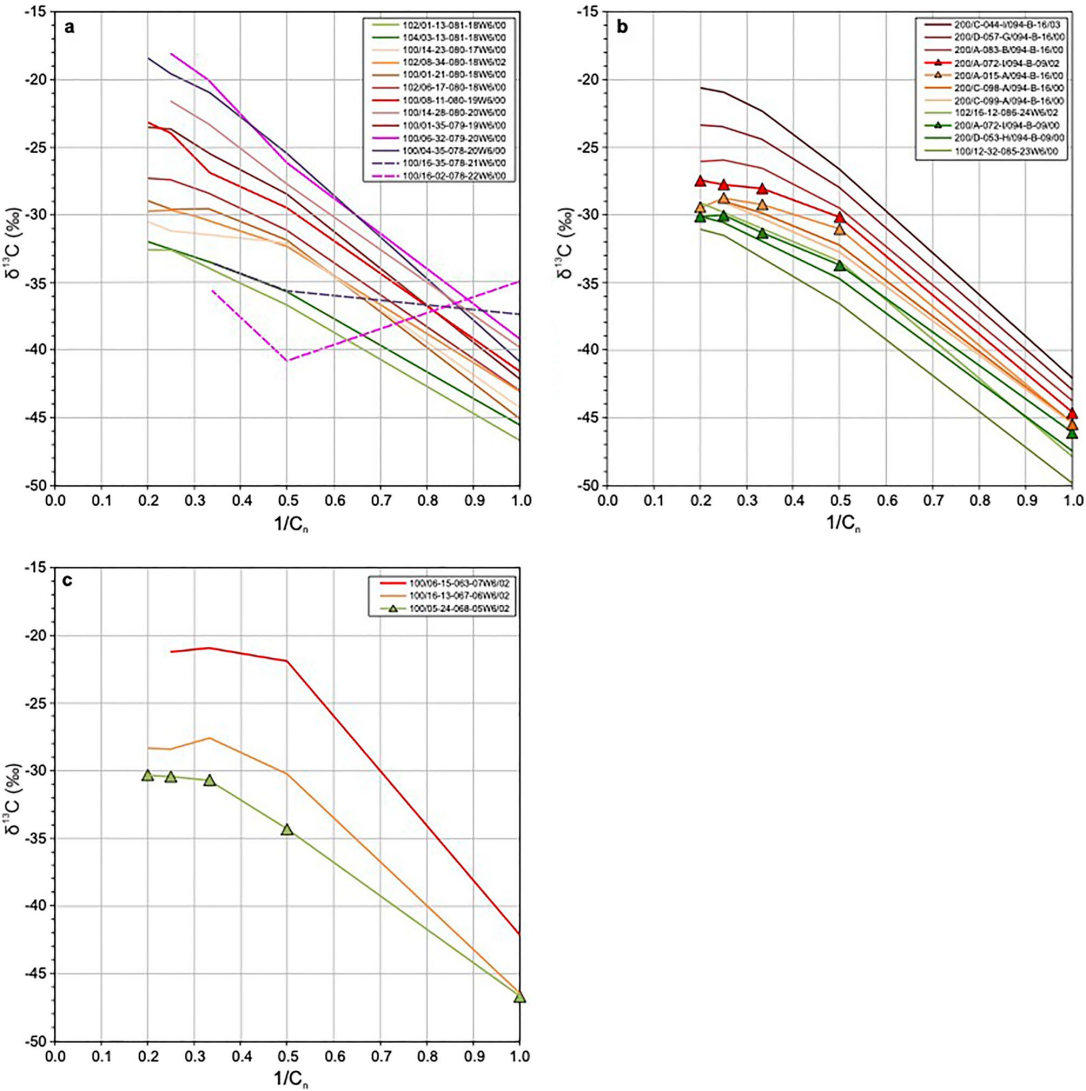
Chung plots^53^ of carbon isotope ratio data for Montney produced gas samples. (**a**) Tower-Groundbirch area. (**b**) North Montney area. (**c**) Gold Creek area. Dashed lines in panel (**a**) delineate gas samples from an isotopic rollover/reversal zone^51,52^. All the other gas samples have normal isotopic signatures^51,52^. Triangles in panels (**b**,**c**) indicate samples with co-produced hydrocarbon liquid data shown in Fig. 5.

Figure 4 presents Chung plots^53^ of reciprocal carbon number vs. carbon isotope ratio for produced gas samples from the three study areas. The plot for the Tower-Groundbirch area (Fig. 4a) shows 13 gas samples representative of the produced fluid maturity range in this area. Two samples from wells located furthest to the southwest (dashed lines) are consistent with isotopic rollover and reversal that was noted in Fig. 3c,d: one well (100/16-02-078-22W6/00) has a partially reversed signature and the other well (100/16-35-078-21W6/00) has an anomalously shallow slope. The other 11 samples have normal isotopic signatures. The lowest maturity sample, from the late oil window (well 102/01-13-081-18W6/00), and the three highest maturity samples (wells 100/14-28-080-20W6/00, 100/06-32-079-20W6/00, 100/04-35-078-20W6/00), from the wet to dry gas windows, have nearly straight-line profiles whereas the intermediate maturity samples all have convex upward profiles.

The Chung plot for the North Montney area (Fig. 4b) shows that all 11 gas samples have normal isotopic signatures in the C_1_–nC_4_ range. The lowest maturity sample, from the late oil window (well 100/12-32-085-23W6/00), has a nearly straight-line distribution whereas all the other samples have convex upward profiles. Some of the isotopic profiles flatten or even reverse slightly forming an isotopic maximum at nC_4_ (e.g., well 200/A-015-A-094-B-16/00). The three highest maturity samples, from the wet gas window, have progressively increasing C_2_–nC_5_ slopes, and the highest maturity sample (well 200/C-044-I/094-B-16/03) has an almost straight line C_1_–nC_5_ distribution like the highest maturity samples at Tower–Groundbirch (Fig. 4a).

The Chung plot for the Gold Creek area (Fig. 4c) shows all three gas samples exhibit convex upward profiles. Major inflections occur at C_3_ in the lowest maturity sample (green line), and at C_2_ in the highest maturity gas sample (red line). The middle maturity sample (orange line) has a major inflection point at C_3_ and a lesser inflection at C_2_. The highest and intermediate maturity samples both have an isotopic maximum at C_3_ and a reversed C_3_–nC_4_ slope.

### Carbon isotope ratios of co-produced gas and hydrocarbon liquid

**Figure 5:**
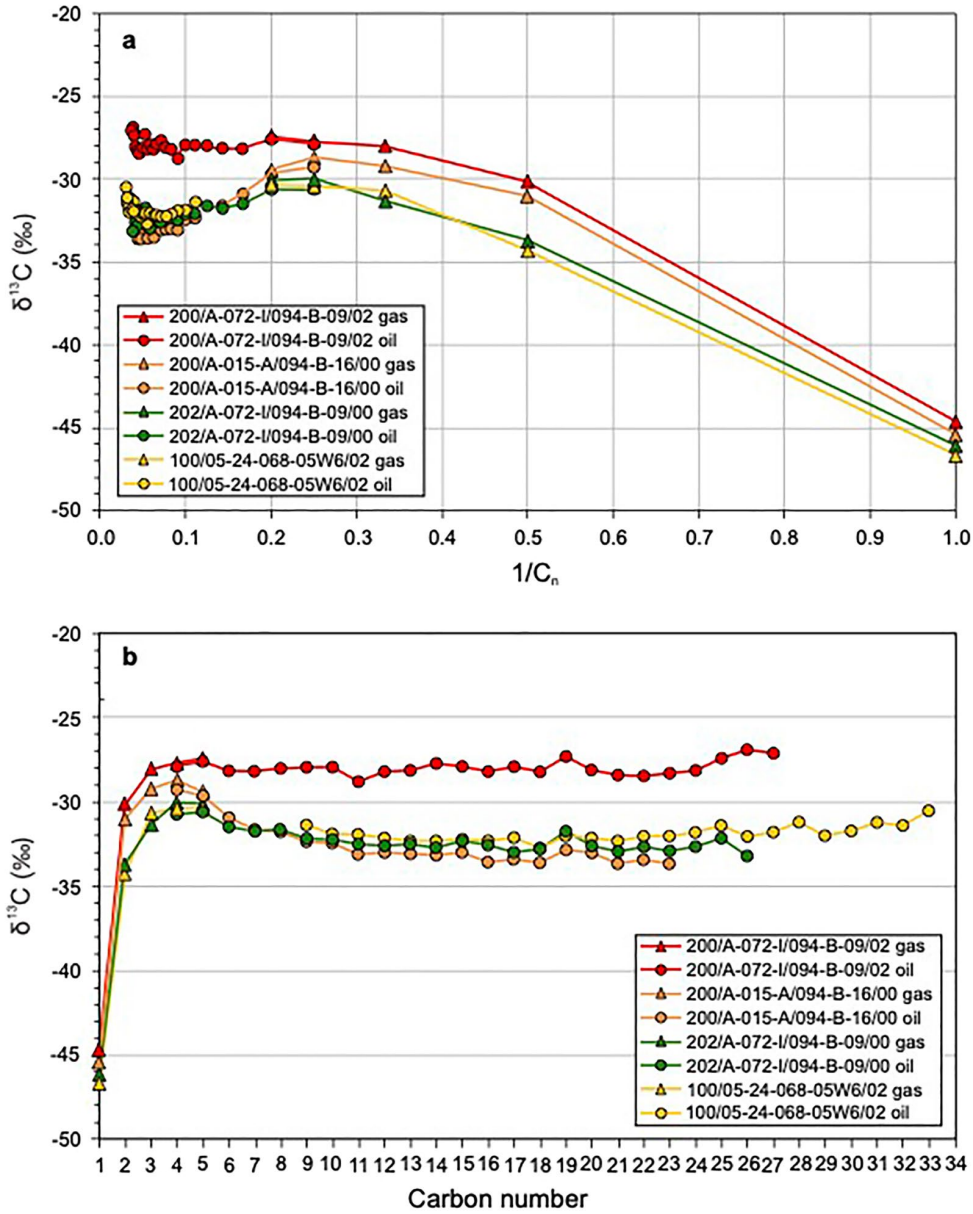
Extended carbon isotope ratio data for four Montney wells with co-produced gas and hydrocarbon liquid data. (**a**) Cross-plot of reciprocal carbon number vs. carbon isotope ratio (Chung plot)^53^. (**b**) Cross-plot of carbon number vs. carbon isotope ratio. Red, orange and green lines indicate wells from the North Montney area. Yellow line indicates a well from the Gold Creek area. Triangles indicate gas samples. Circles indicate hydrocarbon liquid samples. The isotopic profiles progress from a convex upward (“hump”) signature at low molecular weight to an increasing or nearly flat signature at high molecular weight, and suggest mixing of in-situ oil (C_16+_ range) and migrated high-maturity gas-condensate (C_1_–C_15_ range).

The four wells with gas samples indicated by triangles in Fig. 4b,c were also sampled for carbon isotope ratio analysis of co-produced hydrocarbon liquids. Supplementary Table S3 lists the carbon isotope ratio data of the produced hydrocarbon liquid samples from these wells: three from the North Montney area and one from the Gold Creek area (Fig. 1a). Figure 5 shows compound-specific isotope analysis (CSIA) data for both co-produced gas (triangles) and hydrocarbon liquid (circles). The data are presented as a Chung plot with reciprocal carbon number vs. carbon isotope ratio in Fig. 5a, and a cross-plot of carbon number vs. carbon isotope ratio in Fig. 5b. The latter figure helps to clearly show carbon isotope ratios at high carbon numbers, which otherwise cluster closely together on a Chung plot at their corresponding low reciprocal carbon numbers. Co-produced gas and hydrocarbon liquid samples have overlapping and closely matched C_4_ and C_5_ δ^13^C values in each of the three North Montney wells (red, orange and green lines). In combination, the separate gas and hydrocarbon liquid analyses provide a full carbon isotope ratio profile of the produced hydrocarbon fluid of a well from C_1_ to high carbon number (maximum of C_33_ in well 100/05-24-068-05W6/02, yellow line).

On both the Chung plot (Fig. 5a) and the carbon number vs. carbon isotope ratio plot (Fig. 5b), each well profile has a convex upward (“hump”) signature at low molecular weight. The three lower maturity wells (yellow, orange and green lines) have well developed convex upward signatures from C_1_ to about C_16_ to C_18_ with maxima at C_4_ or C_5_. The highest maturity well (red line) has a less pronounced convex upward signature and shows an increase in δ^13^C values from C_1_ to C_5_ followed by a slight decrease from C_5_ to C_6_. The Gold Creek well (yellow line) has the highest analyzed carbon number and the clearest isotopic signature at high molecular weight: δ^13^C values have an increasing trend from about C_18_ to C_33_. Although the profiles of the two lower maturity wells from the North Montney area (orange and green lines) do not extend to as high carbon numbers, their δ^13^C values at high molecular weight are similar to those of the Gold Creek well. The highest maturity well (red line) shows a slight increasing trend in δ^13^C values from C_24_ to C_27_ but otherwise has a nearly uniform (flat) isotopic profile at high molecular weight.

### Recombination PVT compositional data

PVT data were used in this study to characterise the chemical composition of the petroleum fluid present in Montney reservoir zones. Co-produced oil and gas samples were recombined following standard petroleum engineering protocols in the laboratory using appropriate reservoir temperature, reservoir pressure and producing gas to oil ratio. Standard PVT analyses provide individual n-alkane concentrations for methane to pentane, and pseudo-component data at higher carbon numbers. A pseudo-component in the employed gas-chromatographic methodology is a group of compounds that ideally all have the same carbon number. In practice, following Thompson^1,2,54^, the light cyclic compounds were rigorously assigned by carbon number to their appropriate pseudo-component (regardless of actual elution incongruities) whereas beyond pseudo-component 10 (P10) all compounds that eluted immediately after a particular n-alkane were assigned to the following higher n-alkane. The molar concentrations of pseudo-components in unmodified reservoir fluids typically form exponential progressions that decrease with increasing carbon number. The rate of molar concentration change reflects the degree of cracking and thus the slopes of such progressions can be used to assess the thermal maturity levels of reservoir fluids^1,2,54^.

**Figure 6:**
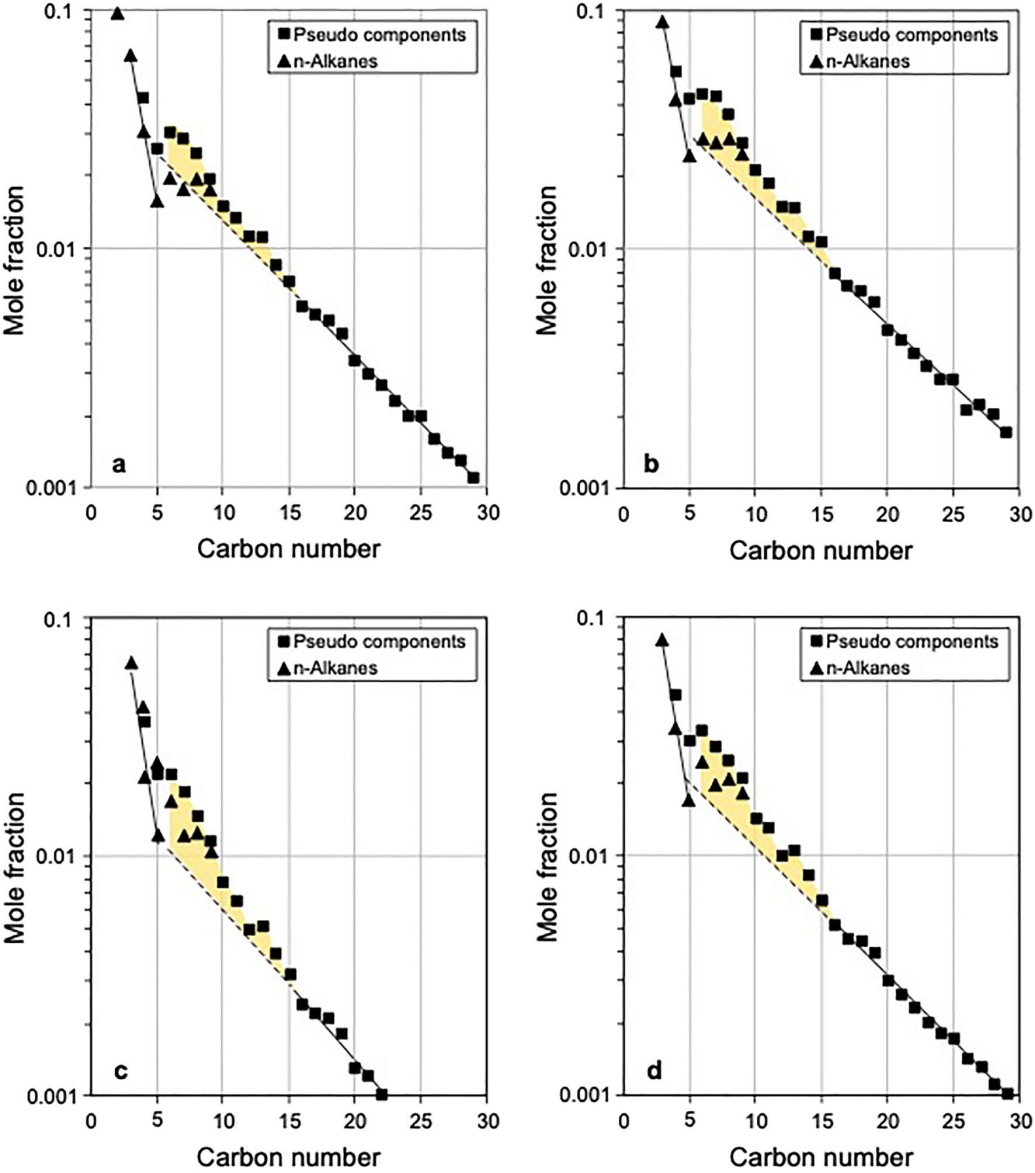
Carbon number vs. mole fraction plots for four Montney wells with recombination PVT data. (**a**) Well 100/16-23-081-18W6/00, Tower area. (**b**) Well 100/12–04-082-18W6/02, Tower area. (**c**) Well 200/A-006-D/094-A-13/02, North Montney area. (**d**) Well 100/07-35-086-23W6/02, North Montney area. Dashed lines indicate projections of C_16+_ trends to lower carbon numbers, and show the hypothetical compositions of original oils prior to subsequent modification of the lighter components^1,2^. The molar concentration profiles all exhibit higher concentrations of pseudo-components in the C_6_–C_15_ gas-condensate range than expected for unmodified original oils (orange shading), and are interpreted to signify mixing of in-situ oil (C_16+_ range) and migrated high-maturity gas-condensate (C_1_–C_15_ range)^1,2^.

Montney recombination PVT compositional data for four wells that offset wells with carbon isotope ratio data are listed in Supplementary Table S4. Two wells are from the Tower area and two are from the North Montney area (Fig. 1a). The recombined compositional data are presented as molar concentration profiles (carbon number vs. mole fraction plots) in Fig. 6. The molar concentration profiles of all wells have decreasing exponential trends in the C_16+_ range of pseudo-components (solid lines, Fig. 6). Projection of the C_16+_ slope to lower carbon numbers (dashed lines, Fig. 6) indicates the hypothetical composition of an original oil prior to any subsequent natural modification of the lighter components^1,2^. All four wells exhibit concentrations of pseudo-components in the C_6_–C_15_ range higher than expected for unmodified original oils (orange shading, Fig. 6).

## Discussion

Montney produced gas samples from the three widely separated geographic areas in this study (Fig. 1a) form a coherent maturity trend of oil-associated thermogenic gases (Fig. 3a), consistent with their generation via secondary cracking of oil and wet gas (Fig. 3b). The two highest-maturity gas samples, from the furthest southwest portion of the Groundbirch area (Fig. 1a), exhibit rollover and reversal of δ^13^C ethane values^51,52^, whereas all the other gas samples comprise a normal thermal maturity trend^51,52^ with δ^13^C ethane values that increase as gas wetness decreases (Fig. 3c), and positively correlated δ^13^C methane and δ^13^C ethane values (Fig. 3d). Isotope rollovers and reversals are globally common in gases from tight-petroleum systems that are late-mature (vitrinite reflectance > 2%) and have undergone significant basin uplift (> 2 km)^52^. The two Montney gas samples with isotope rollover and reversal are from the deepest portion of the Groundbirch area, in a setting that matches the common global correlation of rollover and reversal with high maturity and significant uplift^30^. Although isotope rollovers and reversals like those in the deep Montney at Groundbirch can form through many different geochemical processes, they appear to most commonly result from molecular and isotopic fractionation accompanying differential gas desorption during basin uplift and depressurization^52^.

Isotopic profiles of these oil-associated thermogenic gases on Chung plots (Fig. 4) have similar characteristics in the three different geographic study areas. The Chung plots for the Tower-Groundbirch and North Montney areas (Fig. 4a,b) suggest that gas samples in both areas form an overall progression from straight isotopic profiles at low maturity to convex upward profiles at intermediate maturity, and a return to straight profiles at high maturity. A straight-line profile on a Chung plot indicates a minimally altered, primary thermogenic gas, and reflects kinetic isotope effects during progressive thermal cracking of hydrocarbons generated from a single organic-rich source rock^53,55^. The straight-line profiles in Fig. 4a,b suggest that minimally altered thermogenic gases exist at both low maturity (late oil window) and high maturity (wet to dry gas windows). Chung et al.^53^ originally suggested that convex upward profiles signify mixing of hydrocarbons generated from different source rocks. Subsequently, various other interpretations for convex upward profiles have been proposed including mixing of gases of different maturities^56,57^, secondary cracking of hydrocarbon gas^58,59^, thermochemical sulfate reduction^59^, mixing of thermogenic gas with biogenic gas^59,60^ and biodegradation^61,62^. Some of these mechanisms can be ruled out for the Montney unconventional petroleum system. For example, the deep basin setting has unfavourable conditions for biodegradation and biogenic gas generation including high temperature, hypersaline formation water, and regionally pervasive hydrocarbons rather than water. However, interpretation of convex upward profiles on Chung plots derived solely from gas data can be ambiguous because of the limited range of components, typically C_1_ up to C_5_.

Extended carbon isotope ratio profiles compiled using compound-specific isotope analysis data from co-produced gas and hydrocarbon liquids, such as the four profiles in this study (Fig. 5), help to clarify the interpretation of convex upward profiles observed on gas-only Chung plots. On both the Chung plot (Fig. 5a) and the corresponding carbon number vs. carbon isotope ratio plot (Fig. 5b), each extended profile has δ^13^C values that increase from C_1_ to a maximum at C_4_ or C_5_ and then decrease either significantly (yellow, green and orange samples) or slightly (red sample). It is thus evident that the convex upward profiles on the gas-only Chung plots (Fig. 4) show only the low carbon number range of convex upward “hump” profiles, which are fully expressed in the extended Chung plot (Fig. 5a). The δ^13^C values of the three lower maturity samples (yellow, green and orange lines, Fig. 5) decrease from C_5_ to around C_16_ to C_18_ and then generally increase to the highest analyzed carbon number at C_33_. The extended isotope profiles of these samples thus exhibit a convex upward signature at low molecular weight (from C_1_ to about C_16-18_ with a maximum at C_4_ or C_5_) transitioning to an increasing carbon isotope ratio signature at high molecular weight (from about C_16-18_ to C_33_). In the case of the highest maturity well from the North Montney area (red sample), the transition from convex upward signature at low molecular weight to nearly uniform (flat signature) at high molecular weight appears to be at C_11_ but is difficult to determine unambiguously.

In their study of Montney produced condensates Cesar et al.^33^ did not show extended carbon number vs. carbon isotope ratio profiles using data from co-produced gas and liquid samples, but their C_8+_ profiles for hydrocarbon liquids are similar to those reported here. They attributed such isotopic profiles to the mixing of two approximately contemporaneous condensate charges: (1) “normal condensate” (C_8_–C_20_ range) which has a decreasing or uniform (flat) δ^13^C signature and was generated from in-situ kerogen cracking; (2) “thermal condensate” (C_20_–C_26_ range) which has an increasing δ^13^C signature and was produced by cracking of oil that migrated from adjacent source rocks. PVT data in our study, however, suggest an alternative mixing mechanism for extended isotopic profiles that exhibit a convex upward “hump” signature at low molecular weight and an increasing carbon isotope ratio signature at high molecular weight.

Recombination PVT compositional data from all four studied Montney wells show C_6_–C_15_ condensate range concentrations are higher than expected for unmodified original oils^1,2^ (orange shading, Fig. 6). The carbon number range of excess molar concentration (C_6_–C_15_) closely matches that of the convex upward isotopic signature (C_1_ to about C_16–18_) observed on the Chung plot (Fig. 5a) and corresponding carbon number vs. carbon isotope ratio plot (Fig. 5b). This suggests that the characteristic extended isotopic profiles (progressing from a convex upward signature at low molecular weight to an increasing carbon isotope ratio signature at high molecular weight) are mixing profiles formed by the migration of high-maturity gas-condensate (C_1_–C_15_ range) from deep zones into shallower Montney oil zones with in-situ hydrocarbon liquids of lower thermal maturity. This interpretation accounts for both the excess molar concentrations and the enriched/heavier isotopic signatures of gas-condensate range components compared with an unmodified original oil. The observation that the isotopic profiles with convex upward signature at low molecular weight and increasing carbon isotope ratio signature at high molecular weight are almost identical for wells from the Gold Creek (yellow line, Fig. 5) and North Montney (green line, Fig. 5) areas, which are located 294 km apart (Fig. 1a), suggests that the mechanisms of both original oil charging and later migration of gas-condensate were similar in widely separated areas of the Montney tight-petroleum system.

**Figure 7:**
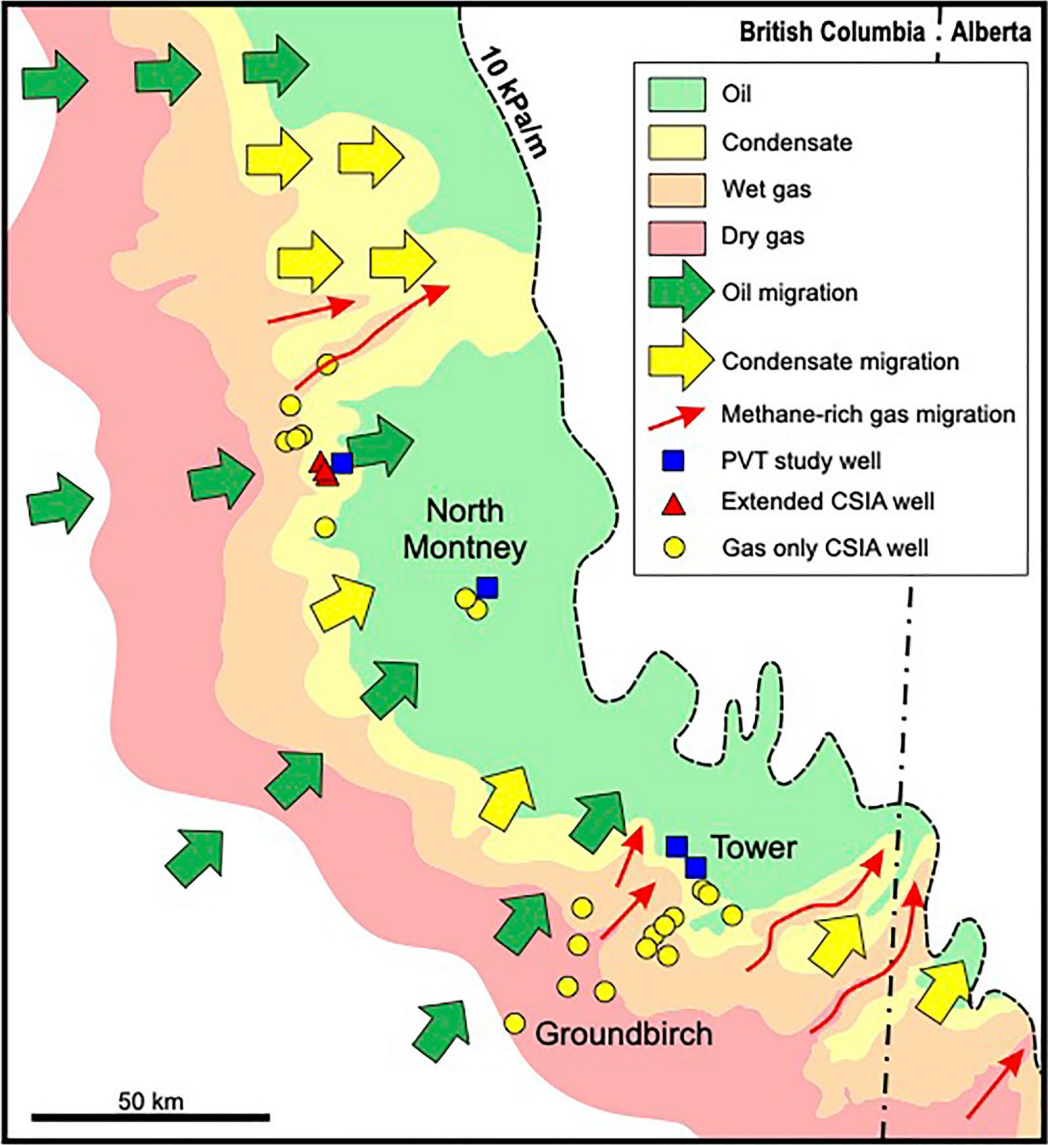
Map schematically showing multiple episodes of internal hydrocarbon migration in the unconventional Montney petroleum system of northeast British Columbia (BC) and northwest Alberta (AB). Colour shaded areas indicate present-day hydrocarbon fluid windows in the Upper Member of the Montney Formation. Green arrows show regionally widespread early migration of oil from distal Montney organic-rich source rocks^18–20^. Yellow arrows show regionally widespread migration of gas-condensate (this study). Red arrows show major pathways of late-stage methane-rich gas migration largely during basin uplift^15,17^. Dashed line indicates 10 kPa/m pressure-depth gradient contour, which marks the approximate updip edge of the over-pressured Montney unconventional petroleum system. Symbols show locations of wells with data used in this study area; *PVT* recombination PVT compositional data, *CSIA* compound-specific isotope analysis. Modified from Euzen et al.^12^. Used with permission of AAPG.

The recognition of widespread gas-condensate migration in this study adds to the complex history of multiple episodes of internal (intraformational) hydrocarbon migration that is emerging for the Montney unconventional petroleum system (Fig. 7), including an early oil migration episode during burial (green arrows)^18–20^, and a late methane-rich gas episode (red arrows)^9–13^ largely during basin uplift^15–17^ (Fig. 2). Gas-condensate generation mostly post-dated the early episode of oil migration, and occurred when increased burial depths and temperatures led to thermal cracking of the originally migrated oil. Internal migration of gas-condensate on a regional scale was driven by the significant pressure difference that existed between deeper and shallower Montney zones. Gas-condensate migrated preferentially to oil due to its much lower viscosity (by one to two orders of magnitude)^8^. The degree of gas-condensate migration was likely not regionally uniform (Fig. 7, yellow arrows) and appears to have extended further up-dip in part of the North Montney area^12^. The migration of late-stage methane-rich gas is thought to have been influenced by both stratigraphic and structural trends^13,15–17^ and a similar interpretation is likely applicable to the earlier migration of gas-condensate. Secondary migration of gas-condensate in the WCSB, it should be noted, is not unique to the unconventional petroleum system of the Triassic Montney Formation, and is known to have occurred in many conventional reservoir zones from Devonian to Cretaceous in age^1,2^.

In the context of the Montney hydrocarbon generation and migration scenarios discussed above, it is worth drawing important distinctions between thermal gas-condensate and hybrid gas-condensate^63^. Gas-condensate that was generated following the early episode of oil migration can be classified as thermal gas-condensate; that is, it formed as part of the natural evolution of a single original petroleum during progressive thermal maturation^63^. In thermal gas-condensate, the gaseous and liquid fractions have equal maturity levels, and will plot together as a coherent straight-line isotopic profile on a Chung plot. The subsequent migration of thermal gas-condensate and its ultimate mixing with shallower Montney oil led to some of the in-situ liquid oil being vaporized into the gaseous phase. The resulting mixed and modified hydrocarbon fluid can be classified as hybrid gas-condensate^63^. In hybrid gas-condensate, the gaseous fraction has a higher maturity level than the liquid fraction, resulting in a Chung plot isotopic signature that deviates from a straight line. The Chung plot of Montney co-produced hydrocarbon gas and liquid (Fig. 5a) exhibits complex isotopic profiles that deviate significantly from straight-lines. Moreover, the combined isotopic profiles have convex upward signatures at low molecular weight and increasing or uniform isotopic signatures at high molecular weight, consistent with the samples being representative of in-situ hybrid gas-condensate in the subsurface.

Laycock et al.^19^ and Watt et al.^20^ observed that the stable carbon isotopes of oil samples extracted from Montney cores have depth trends that can be correlated consistently between wells, and also with global inorganic stable carbon isotope signatures. Because Montney rocks generally contain low amounts of organic matter, the consistent isotopic depth trends are thought to be indicative of internal, bed-parallel migration of oil from an as yet unrecognized deeper Montney source to the southwest. Although present-day Montney rocks are tight with low permeability, lateral migration of oil is conceivable prior to the formation of pore-occluding solid bitumen^32,39–43^ when Montney rocks had significantly higher permeability^42,43^. The dominantly lateral migration of oil through highly laminated rocks was previously considered to have minimized any vertical mixing and homogenization of hydrocarbons, and resulted in a vertically stratified oil column that influences present-day production yields of hydrocarbon gas and liquid^19,20^. Our study results, however, indicate that vertical stratification of Montney oil likely applies only to C_16+_ components, and not to lighter gas-condensate range components, which migrated later with significant bedding-perpendicular transmission that enabled fluid mixing. The latter interpretation suggests that present-day production yields of hydrocarbon gas and liquid (e.g., gas-oil or condensate-gas ratio), are likely to be influenced more by the amount of gas-condensate introduced to an original oil via internal migration than by the vertical stratification of originally migrated C_16+_ oil components.

The multiple episodes of petroleum fluid migration elucidated here for the Montney Formation are components of the long-term global carbon cycle^64,65^ and provide examples how organic carbon fixed deeply in sedimentary basins can be remobilized and transmitted upward through tight (low-permeability) rocks. Migration and leakage of gas from tight unconventional basins, such as the Montney^15–17^, leads to the up-dip transfer of methane from deep accumulations of organic carbon to shallower zones, and ultimately the atmosphere with significant natural greenhouse gas emissions^66,67^.

## Conclusions

Stable carbon isotope and PVT data from co-produced hydrocarbon gas and liquid samples provide evidence of widespread migration of gas-condensate in the early Triassic Montney tight-petroleum system of western Canada. Montney produced gas samples from three widely separated geographic areas form a coherent maturity trend of oil-associated and late mature thermogenic gases. Compositional and isotopic characteristics indicate the two highest-maturity gas samples are from an isotopic rollover/reversal zone, whereas the other 25 gas samples comprise a normal thermal maturity trend. On Chung plots of reciprocal carbon number versus carbon isotope ratio^53^, the normal maturity gas samples show a progression from straight signatures at low maturity to convex upward signatures at intermediate maturity, and a return to straight signatures at high maturity. The straight-line signatures indicate minimally altered, primary thermogenic gases, and reflect kinetic isotope effects during progressive thermal cracking of hydrocarbons^53,55^. The convex upward signatures indicate altered gases^53,55^, but the geologic processes responsible for alteration are ambiguous based solely on the limited carbon number range (C_1_–C_5_) of the gas isotope data.

Extended carbon isotopic ratio profiles (C_1_–C_33_ maximum), using co-produced hydrocarbon gas and liquid samples from four wells, shed light on the convex upward profiles observed on the gas-only Chung plots. The extended isotopic profiles have convex upward “hump” signatures at low molecular weight, with maxima at C_4_ or C_5_, and increasing or nearly uniform carbon isotope ratio signatures at high molecular weight. Recombination PVT compositional data for four wells located close to wells with extended carbon isotope ratio profiles all have concentrations of C_6_–C_15_ components that are higher than expected for unmodified original oils^1,2^. The C_6_–C_15_ range of excess molar concentration closely matches that of the isotopic convex upward signature (C_1_ to about C_16–18_) observed on Chung plots. The combined convex upward signatures (at low molecular weight) and increasing or uniform isotopic signatures (at high molecular weight) are interpreted as mixing profiles produced by the migration of high-maturity gas-condensate (C_1_–C_15_ range) into shallower Montney zones with in-situ hydrocarbon fluids (C_16+_ range) of lower thermal maturity.

The recognition of widespread gas-condensate migration adds to the complex history of internal (intraformational) hydrocarbon migration within the Montney tight-petroleum system including previously identified migration episodes of early oil^18–20^ and late-stage methane-rich gas^15,17^. Multiple episodes of internal hydrocarbon migration are likely to have occurred in many other unconventional tight-petroleum systems globally, and thus the Montney Formation provides a well-documented analogue for comparison.

## Methods

Publicly available carbon isotope ratio data for 27 gas samples and four hydrocarbon liquid samples from wells producing from the Montney Formation were accessed for the study from archives of provincial regulatory authorities (Alberta Energy Regulator and British Columbia Oil and Gas Commission). The gas and liquid samples were studied using compound-specific isotope analysis (CSIA). This analysis uses gas chromatography to separate the constituent compounds, which then go through a combustion unit for conversion to carbon dioxide (CO_2_). The resultant CO_2_ gas is then introduced directly into a mass spectrometer. Computer software is used for peak detection and quantification. δ^13^C values of the species are expressed in per mil (‰) relative to the international V-PDB standard with a typical precision of ± 0.3 ‰. Publicly available Montney PVT studies were accessed from archives of the British Columbia Oil and Gas Commission and four representative wells were selected for presentation here. In PVT studies, well-separator gas and oil samples are physically recombined to characterize in-situ reservoir fluids at appropriate pressure and temperature conditions. Physical recombination of separator gas and liquid samples is performed at a specified gas–liquid ratio in a high-pressure PVT cell. The gas and liquid samples are compositionally analyzed using gas chromatography methods. Recombination PVT data typically provide fluid composition from methane through C_30+_ as well as concentrations of cyclic compounds. Compositions of recombined reservoir fluids were used to prepare molar concentration profile plots (carbon number vs. mole fraction) following the protocols and formats of Thompson^1,2,54,63,68^.

## Supplementary Information

Supplementary Information.

## Data Availability

The data that support the findings of this study are available in the Supplementary Information.
