# Supplementary material for: Geochemical evidence for the internal migration of gas condensate in a major unconventional tight petroleum system
Source: Sci Rep. 2022 May 13;12:7931. doi: 10.1038/s41598-022-11963-6 (PMC9106717; doi:10.1038/s41598-022-11963-6)
Supplement: Supplementary file 1 — Supplementary Information. [file 41598_2022_11963_MOESM1_ESM.pdf]

Geochemical evidence for the internal migration of gas condensate in a major unconventional tight petroleum system

James M. Wood, Jaime Cesar, Omid H. Ardakani, Arka Rudra, Hamed Sanei

Supplementary Information

Table S1 Compound-specific carbon isotope ratio data of produced Montney gas samples

Geographic area	Unique well identifier	Latitude, toe of well	Longitude, toe of well	TVD, Top Montney, m	Elevation, Top Montney, m	TVD, toe of well, m	Elevation, toe of well, m	δ13C1 ‰	δ13C2 ‰	δ13C3 ‰	δ13nC4 ‰	δ13nC5 ‰
North Montney	200/A-083-B/094-B-16/00	56.82046	-122.15757	1911.2	-1030	2047.2	-1166	-43.73	-29.5	-26.53	-25.92	-26.02
North Montney	200/D-057-G/094-B-16/00	56.88082	-122.20487	1940.4	-991.3	2120.3	-1171.2	-42.92	-27.98	-24.41	-23.47	-23.33
North Montney	200/A-072-I/094-B-09/02	56.72866	-122.01985	1768.1	-997.9	1955.8	-1185.6	-44.6	-30.1	-28	-27.7	-27.4
North Montney	200/A-015-A/094-B-16/00	56.76089	-122.05165	1769.8	-999.6	1894.2	-1124	-45.4	-31	-29.2	-28.7	-29.4
North Montney	202/A-072-I/094-B-09/00	56.72862	-122.01978	1768.1	-997.9	1783.6	-1013.4	-46.1	-33.7	-31.3	-30	-30.1
North Montney	200/D-053-H/094-B-09/00	56.63142	-122.03062	2069.8	-1176.2	2075	-1181.4	-47.45	-34.71	-31.98	-30.53	-30.06
North Montney	200/C-044-I/094-B-16/03	56.95557	-122.04841	1902.7	-897.9	2126.8	-1122	-42.05	-26.62	-22.3	-20.9	-20.58
North Montney	102/16-12-086-24W6/02	56.44721	-121.64352	2015.6	-1112.4	2163	-1259.8	-47.86	-33.42	-30.97	-29.79	-29.07
North Montney	100/12-32-085-23W6/00	56.41466	-121.6094	2015.7	-1112.5	2062.9	-1159.7	-49.82	-36.56	-33.05	-31.48	-31.04
North Montney	200/C-099-A/094-B-16/00	56.83035	-122.10943	1936.9	-989.2	1964.2	-1016.5	-45.45	-32.75	-30.21	-29.08	
North Montney	200/C-098-A/094-B-16/00	56.83218	-122.10024	1938.6	-990.9	1956.2	-1008.5	-45.42	-32.22	-29.84	-28.99	
Tower-Groundbirch	102/01-13-081-18W6/00	56.01577	-120.66411	2066.8	-1224.1	2184.7	-1342	-46.7	-36.7	-34	-32.6	-32.6
Tower-Groundbirch	104/03-13-081-18W6/00	56.01502	-120.68122	2047.7	-1242.7	2045.8	-1240.8	-45.6	-35.7	-33.5	-32.6	-32
Tower-Groundbirch	100/14-23-080-17W6/00	55.95475	-120.54594	2002.8	-1283.1	2119.2	-1399.5	-44.3	-32.1	-31.5	-31.2	-30.5
Tower-Groundbirch	102/08-34-080-18W6/02	55.88124	-120.74301	2131	-1305.1	2207.4	-1381.5	-43.08	-32.36	-30.41	-29.67	-29.74
Tower-Groundbirch	100/01-21-080-18W6/00	55.94461	-120.7434	2225.4	-1374.6	2350.7	-1499.9	-45.1	-31.9	-29.6	-29.6	-29
Tower-Groundbirch	102/06-17-080-18W6/00	55.93091	-120.78239	2246.8	-1427.2	2317.1	-1497.5	-43.03	-31.16	-28.44	-27.44	-27.31
Tower-Groundbirch	100/08-11-080-19W6/00	55.91689	-120.84789	2377.4	-1477.8	2412.9	-1513.3	-41.6	-29.5	-26.9	-24	-23.2
Tower-Groundbirch	100/14-28-080-20W6/00	55.96757	-121.06861	2370.1	-1462.3	2380.2	-1472.4	-39.84	-27.77	-23.36	-21.63	
Tower-Groundbirch	100/01-35-079-19W6/00	55.88618	-120.84796	2390.8	-1541	2465.6	-1615.8	-42.2	-28.5	-25.5	-23.7	-23.5
Tower-Groundbirch	100/06-32-079-20W6/00	55.88822	-121.09354	2615.5	-1718.3	2656.7	-1759.5	-39.21	-26.2	-20.16	-18.13	
Tower-Groundbirch	100/04-35-078-20W6/00	55.79868	-120.99557	2604.3	-1855.1	2651.9	-1902.7	-40.9	-25.5	-21	-19.6	-18.4
Tower-Groundbirch	100/16-35-078-21W6/00	55.80802	-121.13029	2756.6	-1947.5	3110.8	-2301.7	-37.36	-35.62	-33.5		
Tower-Groundbirch	100/16-02-078-22W6/00	55.73512	-121.29015	3087.5	-2358.1	3365	-2635.6	-34.91	-40.83	-35.5		
Gold Creek	100/05-24-068-05W6/02	54.89891	-118.63013	2257.9	-1585.6	2360.8	-1688.5	-46.66	-34.3	-30.67	-30.42	-30.32
Gold Creek	100/06-15-063-07W6/02	54.44887	-118.97156	3693.2	-2564.9	3787.1	-2658.8	-42.17	-21.86	-20.95	-21.21	
Gold Creek	100/16-13-067-06W6/02	54.80551	-118.76636	2558.5	-1817.7	2633.8	-1893	-46.45	-30.26	-27.58	-28.4	-28.32

Table S2 Compositional data of produced Montney gas samples

Geographic Area	Unique well identifier	He %	O2+Ar %	CO2 %	N2 %	H2S %	C1 %	C2 %	C3, %	iC4 %	nC4 %	iC5 %	nC5 %	C6+ %	Wetness %	Σ(C2-C5)/Σ(C1-C5)	C1/(C2+C3)
North Montney	200/A-083-B/094-B-16/00			0.14	0.22		85.69	8.93	2.78	0.68	0.76	0.28	0.2	0.27		13.72	7.32
North Montney	200/D-057-G/094-B-16/00			0.22	0.32		88.5	7.62	1.95	0.49	0.44	0.16	0.09	0.15		10.83	9.25
North Montney	200/A-072-I/094-B-09/02		0.91	0.08	2.53		78.2	10.75	4.39	0.95	1.3	0.36	0.28	0.25		18.74	5.17
North Montney	200/A-015-A/094-B-16/00		0.76	0.09	1.75		80.55	10.44	3.89	0.76	1.07	0.27	0.22	0.2		17.13	5.62
North Montney	202/A-072-I/094-B-09/00		0.89	0.11	2.34		78.62	11.13	4.39	0.72	1.13	0.24	0.24	0.2		18.5	5.07
North Montney	200/D-053-H/094-B-09/00			0.12	0.3		79.36	12.15	5.1	0.77	1.31	0.27	0.28	0.31		20.03	4.6
North Montney	200/C-044-I/094-B-16/03		0.11	0.23	0.59		90.05	6.02	1.62	0.51	0.4	0.2	0.09	0.18		8.94	11.79
North Montney	102/16-12-086-24W6/02	0.01		0.09	33.27		51.49	7.98	3.99	0.61	1.37	0.37	0.35	0.47		22.18	4.3
North Montney	100/12-32-085-23W6/00	0.01	0.27	0.2	37.71		47.97	7.55	3.58	0.5	1.13	0.27	0.35	0.46		21.81	4.31
North Montney	200/C-099-A/094-B-16/00		3.11	0.13	10.37		71.15	9.64	3.61	0.61	0.87	0.2	0.18	0.14		17.51	5.37
North Montney	200/C-098-A/094-B-16/00		0.05	0.13	0.25		81.66	11.14	4.16	0.75	1.07	0.28	0.25	0.26		17.77	5.34
Tower-Groundbirch	102/01-13-081-18W6/00		0.16	0.18	0.06		83.32	9.95	4.17	0.52	0.98	0.19	0.22	0.25		16.13	5.9
Tower-Groundbirch	104/03-13-081-18W6/00			0.14	0.04		83.05	10.08	4.46	0.61	1.11	0.19	0.21	0.11		16.71	5.71
Tower-Groundbirch	100/14-23-080-17W6/00			0.06	0.08		83.85	10.72	3.66	0.49	0.69	0.13	0.14	0.2		15.87	5.83
Tower-Groundbirch	102/08-34-080-18W6/02			0.12	0.21		85.04	9.12	3.31	0.57	0.91	0.22	0.25	0.25		14.46	6.84
Tower-Groundbirch	100/01-21-080-18W6/00		0.07	0.12	0.03		86.08	8.8	3.07	0.53	0.77	0.18	0.19	0.16		13.59	7.25
Tower-Groundbirch	102/06-17-080-18W6/00			0.16	2.84		82.03	9.01	2.96	0.54	0.76	0.23	0.24	0.46		14.35	6.85
Tower-Groundbirch	100/08-11-080-19W6/00		0.07	0.24	0.03		90.59	6.39	1.72	0.34	0.36	0.1	0.08	0.08		9.03	11.17
Tower-Groundbirch	100/14-28-080-20W6/00			0.26	0.47		92.55	4.9	1.11	0.25	0.21	0.06	0.04	0.07		6.63	15.4
Tower-Groundbirch	100/01-35-079-19W6/00		0.26	0.21	0.1		89.53	7.11	1.86	0.35	0.36	0.09	0.07	0.07		9.9	9.98
Tower-Groundbirch	100/06-32-079-20W6/00			0.42	0.17		94.96	3.61	0.56	0.12	0.08	0.02	0.01	0.03		4.43	22.78
Tower-Groundbirch	100/04-35-078-20W6/00		0.06	0.31	0.04		94.23	4.27	0.78	0.14	0.11	0.02	0.01	0.01		5.36	18.64
Tower-Groundbirch	100/16-35-078-21W6/00		0.03	2.42	1.44		95.18	0.86	0.05							0.94	105.03
Tower-Groundbirch	100/16-02-078-22W6/00		0.27	5.15	1.24		92.64	0.61	0.06					0.01		0.72	137.55
Gold Creek	100/05-24-068-05W6/02		1.86	0.45	10.31		73.45	7.86	3.71	0.49	1.17	0.24	0.26	0.2		15.75	6.35
Gold Creek	100/06-15-063-07W6/02		0.05	0.33	0.39	13.22	75.26	4.64	2.23	0.99	1.05	0.67	0.45	0.73		11.76	10.95
Gold Creek	100/16-13-067-06W6/02			0.27	10.3		75.51	7.87	3.5	0.56	1.18	0.27	0.28	0.26		15.32	6.64

Table S3 Compound-specific carbon isotope ratio data of produced Montney hydrocarbon liquid samples

Geographic area	North Montney	North Montney	North Montney	Gold Creek
Unique well identifier	200/A-072-I/094-B-09/02	200/A-015-A/094-B-16/00	202/A-072-I/094-B-09/00	100/05-24-068-05W6/02
Latitude, toe of well	56.72866	56.76089	56.72862	54.89891
Longitude, toe of well	-122.01985	-122.05165	-122.01978	-118.63013
True vertical depth, top Montney, m	1768.1	1769.8	1768.1	2257.9
Subsea elevation, top Montney, m	-997.9	-999.6	-997.9	-1585.6
True vertical depth, toe of well, m	1955.8	1894.2	1783.6	2360.8
Subsea elevation, toe of well, m	-1185.6	-1124	-1013.4	-1688.5
δ13C nC4	-27.9	-29.3	-30.7	
δ13C nC5	-27.6	-29.7	-30.6	
δ13C nC6	-28.2	-30.9	-31.5	
δ13C nC7	-28.2	-31.6	-31.8	
δ13C nC8	-28	-31.8	-31.6	
δ13C nC9	-28	-32.4	-32.1	-31.4
δ13C C10	-28	-32.5	-32.2	-31.9
δ13C C11	-28.8	-33.1	-32.5	-31.9
δ13C C12	-28.2	-33	-32.5	-32.1
δ13C C13	-28.1	-33.1	-32.5	-32.3
δ13C C14	-27.7	-33.1	-32.6	-32.3
δ13C C15	-27.9	-33	-32.3	-32.2
δ13C C16	-28.2	-33.6	-32.6	-32.3
δ13C C17	-27.9	-33.4	-33	-32.1
δ13C C18	-28.2	-33.6	-32.8	-32.7
δ13C C19	-27.3	-32.8	-31.7	-32
δ13C C20	-28.1	-33	-32.6	-32.1
δ13C C21	-28.4	-33.7	-32.9	-32.3
δ13C C22	-28.5	-33.4	-32.7	-32
δ13C C23	-28.3	-33.6	-32.9	-32
δ13C C24	-28.1		-32.6	-31.8
δ13C C25	-27.4		-32.2	-31.4
δ13C C26	-26.9		-33.2	-32
δ13C C27	-27.1			-31.8
δ13C C28				-31.2
δ13C C29				-32
δ13C C30				-31.7
δ13C C31				-31.2
δ13C C32				-31.4
δ13C C33				-30.5

Table S4 Compositional data for Montney recombination PVT samples

Geographic area		North Montney	North Montney	Tower	Tower
Unique well identifier		100/07-35-086-23W6/02	200/A-006-D/094-A-13/02	100/12-04-082-18W6/02	100/16-23-081-18W6/00
Latitude, toe of well		56.500715	56.751282	56.082582	56.040925
Longitude, toe of well		-121.515882	-121.942955	-120.764407	-120.690455
True vertical depth, top Montney, m		1806.9	2000	1858.1	2027.4
Subsea elevation, top Montney, m		-1033.1	-1037.7	-1140.8	-1185.8
True vertical depth, toe of well, m		1821.8	2018	1892.3	2035
Subsea elevation, toe of well, m		-1048	-1055.7	-1175	-1193.4
Hydrogen	H				0.0001
Nitrogen	N2	0.0039	0.0031	0.0022	0.0014
Carbon Dioxide	CO2	0.0045	0.001	0.0007	0.002
Hydrogen Sulfide	H2S	0.0001			
Methane	C1	0.5032	0.6434	0.3961	0.5452
Ethane	C2	0.1109	0.1118	0.101	0.0958
Propane	C3	0.0808	0.0648	0.0877	0.0643
i-Butane	i-C4	0.0131	0.0153	0.0134	0.0119
n-Butane	n-C4	0.0341	0.0214	0.0413	0.0307
i-Pentane	i-C5	0.0119	0.0093	0.0152	0.0101
n-Pentane	n-C5	0.0171	0.0123	0.0242	0.0158
Hexanes	C6	0.0247	0.017	0.0286	0.0196
Heptanes	C7	0.0198	0.0122	0.0274	0.0175
Octanes	C8	0.0209	0.0125	0.0285	0.0193
Nonanes	C9	0.0182	0.0104	0.0246	0.0175
Decanes	C10	0.0143	0.0078	0.0211	0.0149
Undecanes	C11	0.0131	0.0065	0.0186	0.0134
Dodecanes	C12	0.0101	0.0049	0.0148	0.0111
Tridecanes	C13	0.0105	0.0051	0.0147	0.0111
Tetradecanes	C14	0.0083	0.0039	0.0112	0.0085
Pentadecanes	C15	0.0065	0.0032	0.0105	0.0073
Hexadecanes	C16	0.0051	0.0024	0.0078	0.0057
Heptadecanes	C17	0.0045	0.0022	0.0069	0.0053
Octadecanes	C18	0.0044	0.0021	0.0066	0.005
Nonadecanes	C19	0.0039	0.0018	0.0059	0.0044
Eicosanes	C20	0.003	0.0013	0.0045	0.0034
Heneicosanes	C21	0.0026	0.0012	0.0041	0.003
Docosanes	C22	0.0023	0.001	0.0036	0.0027
Tricosanes	C23	0.002	0.0009	0.0032	0.0023
Tetracosanes	C24	0.0018	0.0008	0.0028	0.002
Pentacosanes	C25	0.0017	0.0007	0.0028	0.002
Hexacosanes	C26	0.0014	0.0006	0.0021	0.0016
Heptacosanes	C27	0.0013	0.0005	0.0022	0.0014
Octacosanes	C28	0.0011	0.0004	0.002	0.0013
Nonacosanes	C29	0.001	0.0004	0.0017	0.0011
Tricontanes Plus	C30+	0.0117	0.0028	0.0179	0.0116
Cyclopentane	C5H10	0.0012	0.0005	0.0026	0.0001
Methylcyclopentane	C6H12	0.0034	0.0018	0.007	0.0049
Cyclohexane	C6H12	0.0039	0.0024	0.0074	0.005
Methylcyclohexane	C7H14	0.0062	0.0042	0.0122	0.0091
Benzene	C6H6	0.0014	0.0008	0.0012	0.0008
Toluene	C7H8	0.0027	0.0021	0.0032	0.0022
Ethylbenzene & p,m-Xylene	C8H10	0.0025	0.0015	0.006	0.004
o-Xylene	C8H10	0.0017	0.0008	0.0017	0.0017
1,2,4-Trimethylbenzene	C9H12	0.003	0.0011	0.0028	0.0019
